# Supplementary material for: CRISPR Deletion of a SVA Retrotransposon Demonstrates Function as a cis-Regulatory Element at the TRPV1/TRPV3 Intergenic Region
Source: Int J Mol Sci. 2021 Feb 15;22(4):1911. doi: 10.3390/ijms22041911 (PMC7917899; doi:10.3390/ijms22041911)
Supplement: Supplementary file 1 [file ijms-22-01911-s001.zip › Supplementary file 1.docx]

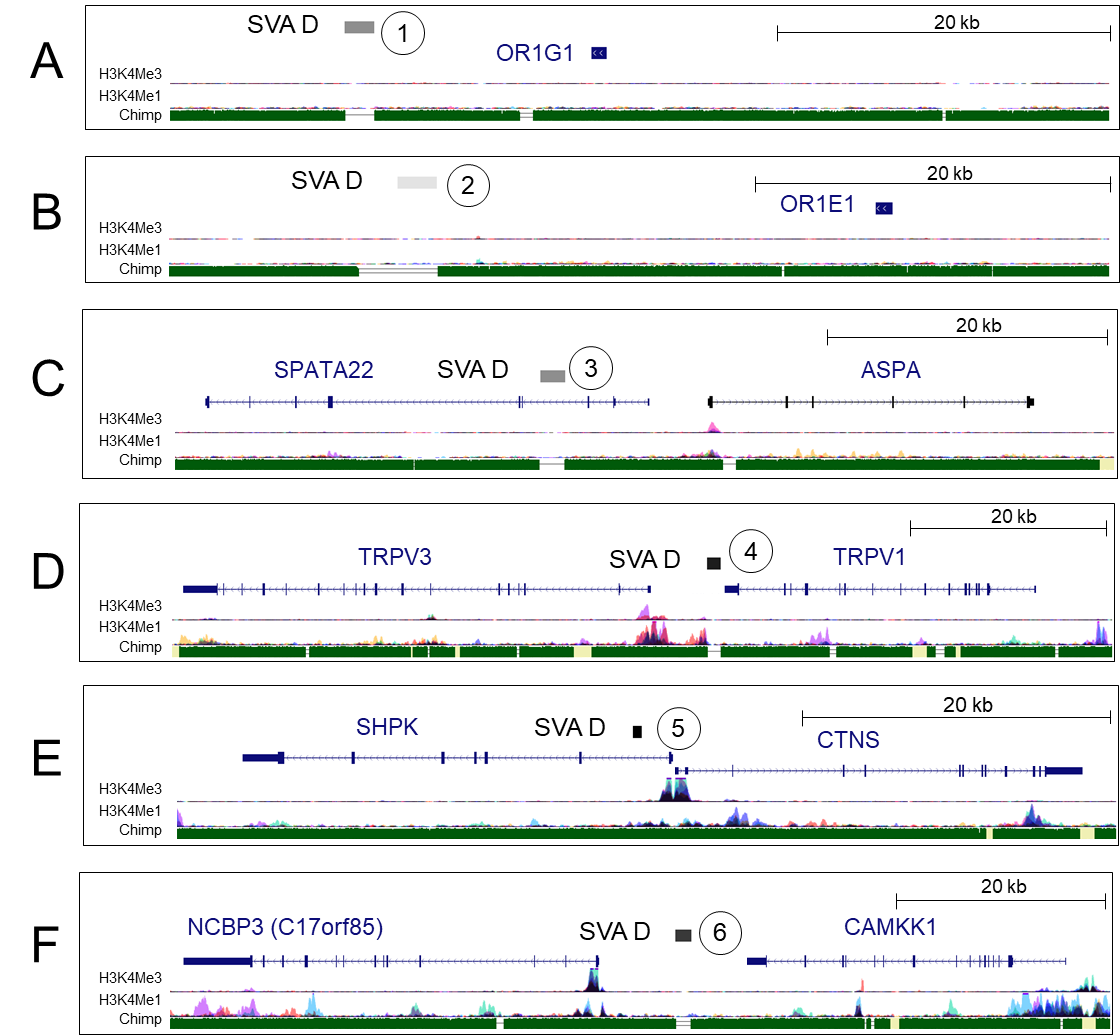


Fig. S1. UCSC screenshots showing overview of SVA insertions at chr17p13.2 with respect to the nearest protein coding genes. (A-F) Labels 1-6 are cross referenced with details shown in fig. 1B. Histone marks H3K4Me3 and H3K4Me1 are indicative of regulatory domains. Sequence conservation with the chimpanzee genome is also shown to highlight human specificity of SVA D insertions across chr17p13.2
