# Supplementary material for: CRISPR Deletion of a SVA Retrotransposon Demonstrates Function as a cis-Regulatory Element at the TRPV1/TRPV3 Intergenic Region
Source: Int J Mol Sci. 2021 Feb 15;22(4):1911. doi: 10.3390/ijms22041911 (PMC7917899; doi:10.3390/ijms22041911)
Supplement: Supplementary file 1 [file ijms-22-01911-s001.zip › Supplementary file 2.docx]

Supplementary file 2. DNA sequences of cloned fragments used in reporter gene assays. Sequences listed below are amplicons produced from PCR and resemble the fragments cloned into reporter gene constructs in their endogenous/forward orientations.

SVA sequence

>chr17:3466879-3468389

GATACTCTACTCTGCTCTCCCTctccctctccctctccctctccctctcc

ctctccccatggtctccctctcccgatggtctccctctccctctctttcc

acggtctccctctcatgccgagccgaagctggactgtactgctgccatct

cggctcactgcaacctccctgcctgattctcctgcctcagcctgcccagt

gcctgcgattgcaggcgcgcgccgccacgcctgactggttttcgtatttt

tttggtggagacgggtttcgctgtgttggccgggctggtctccagctcct

aaccgcgagtgatccgccagcctcggcctcccgaggtgccgggattgcag

acggtgtctggttcactcagtgctcaatggtgcccaggctggagtgcagt

ggcgtgatctcggctcactacaacctccacctcccagccgcctgccttgg

cctcccaaagtgcaaagcttgcagcctctgcctggccgcccatcgtctgg

gatgtgaggagcccctctgcctggctgccccgtctggaaagtgaggactg

tctccgcccggccgcccatcgtctgagatgtggggagcgcctctgccccg

ccgccccatctgggatgtgaggagcgcctctgcccggccacgaccccgtc

tgggaggtgaggagcgtctctgcccagccgccccgtctgagaagtgagga

gcccctccacccggcagctgcccctactgggaagtgaggagcgtctccgc

ccggcagccaccccgtccaggaaggaggtgggggtcacccaccgccaggc

cagccgccccgtccgggaggtgaggggcgcctctgcctggccgcccctac

tgggaagtgaggagcccctctgcccggccaccaccccgtctgggaggtgt

gcccaacagctcattgagaacgggccatgatgacaatggcggttttgtgg

aatagaaaagggggaaaggtggggaaaagattgagaaatcggatggttgc

cgtgtctgcgtagaaagaagtagacatgggagacttcattttgttctgta

ctaagaaaaattcttctgccttgggatcctgttgatctgtgaccttaccc

ccaaccctgtgctctctgaaacatgtgctgtgtccactcagggttaaatg

gattaagggcggtgcaagatgtgctttgttaaacagatgcttgaaggcag

catgctcgttaagagtcatcaccactccctaatctcaagtacccagagac

acaaacactgcggaaggccgcagggtcctctgcctaggaaaaccagagac

ctttgttcacttgtttatctgctgaccttccctccactatcgtcctatga

ccctgccaaatccccctctgtgagaaacacccaagaatgatcaataaaaa

aataaattaaaaaaaaaaagataaaaaataaacacattgtgaaaaaaaaa

aaagaaacagatactctgaaagagagatctgttccagacTGGGCAGGTTG

TGTTAGTATCT

ECR sequence

>chr17:3465700+3466895

ATTGAGCCATGACCACACCactgtgctcccacctgggcaagagagcaaga

ccctgtctcaaaaaaataaaattaaaataaagcttattaattttttaaaa

aagaaagacatatgaattgcaggtatatccttccttaaacaatctcaggt

ttccaatctaataacaaaaaatttctatcattagttttgcaaaagtacaa

ccacaggatcccttccagctctgagtaaatttgaaagctgatccaggctg

ggcacggtggctcacacctgtaatcccagtactttgggaaaccaaggcag

ctggatcacttgaggtcaggagttcaagaccagccccgccaacatggtga

aaccccatttctactaaaactacaaaaattagccaggcgtggtggcaggc

acctgtcatccagctactaaagaggctgaggcaggggaatcccttgaacc

cagaaggcagaggttgcagtgagctgagatcatgccactgcactccagcc

tgggtgacagagcaagactctgtctcaaaaaacaaaacaaaacaaaaagc

tgatccaatttgcagaagtttgcagctgtctgacacccacacattttcta

agaaaggcctacaggtaaagcatggagccccaggcccacagcaggaagcg

agaagccctcccacctccctctggcccaggataggctggcatgccccagc

acattctctgccaggcagcctgacacagcagttaagagccaggctttgga

gtcagactgcctggggtcaaacctaggcaacgtgtttactggctgtgcaa

ccttactcactgttggaaccttgattttctcatctataaaagggaactaa

taatagcttcttcacgagctgttaggagacttaaagggagatgcacatgg

agcaccttcataaacactccccacatcccggctgtcggcctccctcctcc

aggctccaggctcacttgccatagtcacatgcaggcaactggggctcctc

cgtggcgcctcctatgtggaggcagacatgatgctagagaacagggagga

gggatcccagtattttctcccaaaacacctctcctggtagggcccaagcc

tcagcctcagccaaagttcatcctttcatgaaccaacatccaataattcc

acaactgaacaactcattctacactCCGCAGATACTAACACAACCT
