# Supplementary material for: CRISPR Deletion of a SVA Retrotransposon Demonstrates Function as a cis-Regulatory Element at the TRPV1/TRPV3 Intergenic Region
Source: Int J Mol Sci. 2021 Feb 15;22(4):1911. doi: 10.3390/ijms22041911 (PMC7917899; doi:10.3390/ijms22041911)
Supplement: Supplementary file 1 [file ijms-22-01911-s001.zip › Supplementary file 4.docx]

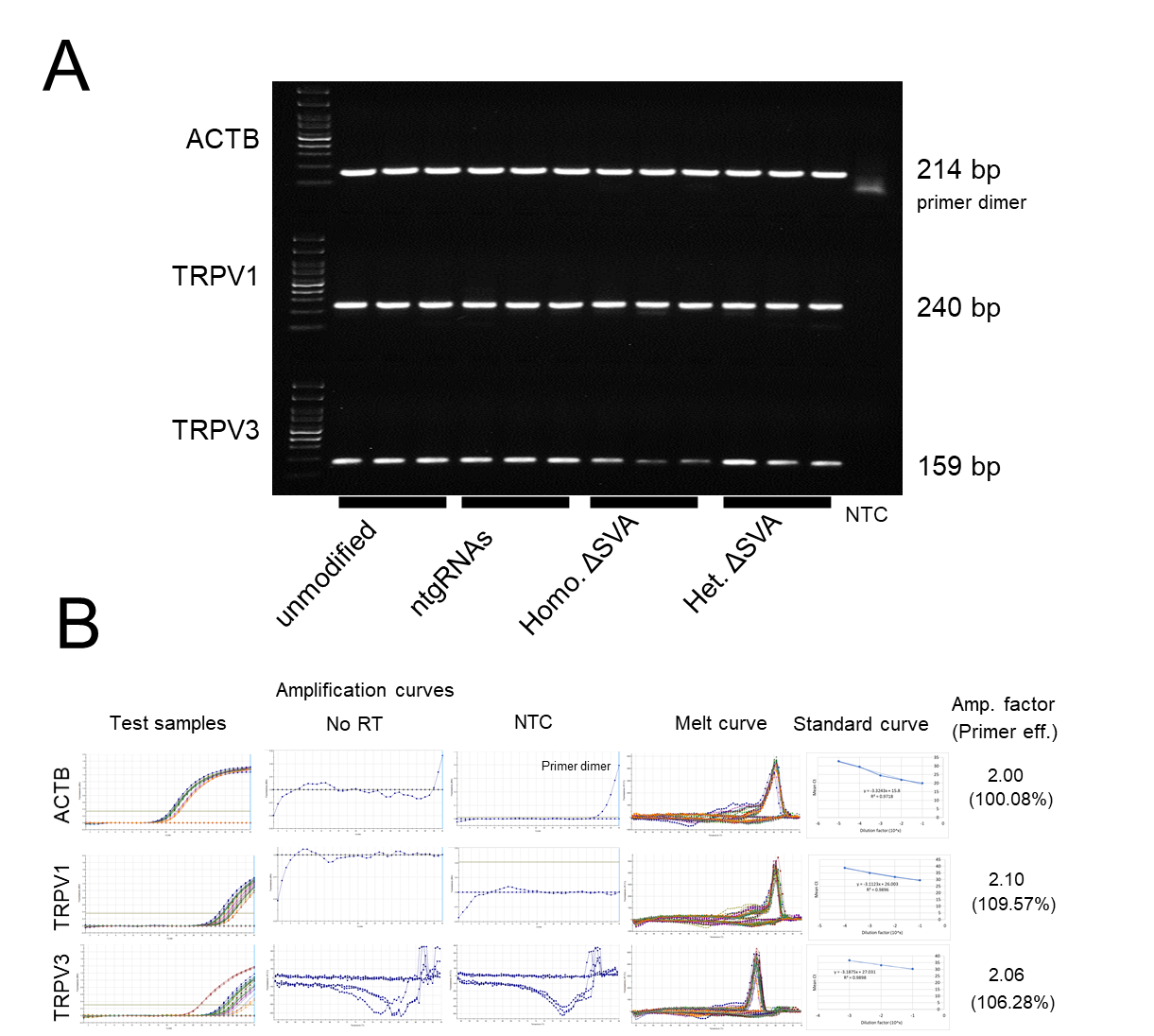


Fig. S2. Validation of RT-PCR used to quantify gene expression. A) RT-PCR of ACTB, TRPV1 and TRPV3 from cDNA prepared unedited, ntgRNA controls and edited HEK293 clonal cell lines; including both homozygous (homo.) and heterozygous (het.) SVA cell lines. No template control (NTC) is also shown. B) Validation of qPCR assay. Amplification curves display real time detection of fluorescence (y-axis) versus cycle number (x-axis). Plots shown include test samples included in study which shows good amplification, no reverse transcription (RT) control showing no amplification, and NTC showing no amplification. Melt curves show a distinct peak in all three reactions, indicating the formation of a single product. Standard curves were plotted using a serial dilution of cDNA template to calculate amplification factor and primer efficiency.
