# Supplementary material for: CRISPR Deletion of a SVA Retrotransposon Demonstrates Function as a cis-Regulatory Element at the TRPV1/TRPV3 Intergenic Region
Source: Int J Mol Sci. 2021 Feb 15;22(4):1911. doi: 10.3390/ijms22041911 (PMC7917899; doi:10.3390/ijms22041911)
Supplement: Supplementary file 1 [file ijms-22-01911-s001.zip › Supplementary file 5.docx]

Supplementary file 5. Sequence reads across breakpoints in PCR products containing SVA alleles following CRISPR modification.

gRNA SNPs no alignment ^=DSB site

>1F

GGAGGGATCCCAGTATTTTCTCCCAAAACACCTCTCCTGGTAGGGCCCAAGCCTCAGCCTCAGCCAAAGTTCATCCTTTCATGAACCAACATCCAATAATTCCACAACTGAACAACTCATTCTACACTCCGCAGATACTAACACAACCTGCCCA^GACTGTACACGCTCTTACACTCACGACGTCATCAAATATGTCAT

>2F

GGAGGGATCCCAGTATTTTCTCCCAAAACACCTCTCCTGGTAGGGCCCAAGCCTCAGCCTCAGCCAAAGTTCATCCTTTCATGAACCAACATCCAATAATTCCACAACTGAACAACTCATTCTACACTCCGCAGATACTAACACAACCTGCCCA^GaCTGGAcaAGATCTCTCTTTCAGAGTATCTGTTTCTTTTTTTTTTTTtCACAcg

>3F

GGAGGGATCCCAGTATTTTCTCCCAAAACACCTCTCCTGGTAGGGCCCAAGCCTCAGCCTCAGCCAAAGTTCATCCTTTCATGAACCAACATCCAATAATTCCACAACTGAACAACTCATTCnACACTCCGCAGATACTAACACtACCTGCCCA^GTCTGGAACAGATCTCTCTTTCAGAGTATCTGTTTCTTTTTTTTTTTT

>1R

AAATGGGCTGGCATAGCTAATTGGCTCTGTGTAGGCTGCTTGTGGATGCGTGTGTATACACATGGGGGTGTGTGTGTGGGTGTGTGCATGCATGCATGTTTGTGTGTGTGTGTGTACGTGTGTGTACATTC^tgggcgggg

>2R

AAATGGGCTGGCATAGCTAATTGGCTCTGTGTAGGCTGCTTGTGGATGCGTGTGTATACACATGGGGGTGTGTGTGTGGGTGTGTGCATGCATGCATGTTTGTGTGTGTGTGTGTACGTGTGTGTACATTC^ngggggggg

>3R

AAATGGGCTGGCATAtCTAATTGGCTCTGTGTAGGCTGCTTGTGGATGCGTGTGTATACACATGGGGGTGTGTGTGTGGGTGTGTGCATGCATGCATGTTTGTGTGTGTGTGTtttCGTGTGTGTACATTC^CTAgGGTTGCgTTCCTGCTAT
