# Supplementary material for: CRISPR Deletion of a SVA Retrotransposon Demonstrates Function as a cis-Regulatory Element at the TRPV1/TRPV3 Intergenic Region
Source: Int J Mol Sci. 2021 Feb 15;22(4):1911. doi: 10.3390/ijms22041911 (PMC7917899; doi:10.3390/ijms22041911)
Supplement: Supplementary file 1 [file ijms-22-01911-s001.zip › Supplementary file 6.docx]

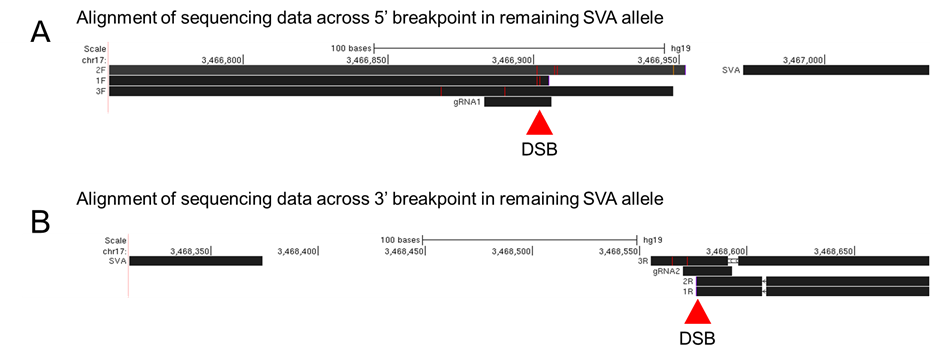


Fig. S3. Sequencing across double strand breakpoints for gRNA1 at the 3’ and gRNA2 at the 5’ end in PCR products containing SVA alleles following CRISPR modification. (A) Sequence alignment of 5’ end across predicted DSB initiated by gRNA1 in clone 1, 2 and 3. Reads for clone 2 and 2 align to hg19 beyond the predicted DSB indicating that the sequence remains intact. Clone 1 sequence does not align with hg19 past the predicted DSB indicating that sequence modification has occurred. (B). Sequence alignment of 3’ end across predicted DSB initiated by gRNA1 in clone 1, 2 and 3. Reads for clone 1 and 2 do not align with hg19 beyond the predicted DSB indicating that sequence modification has occurred. Read for clone 3 aligns with hg19 beyond the predicted DSB indicating that the sequence remains intact. Clone 1 and 2 contain ‘repaired’ SVA alleles with some sequence modifications. Clone 3 remaining SVA allele is unmodified with sequence alignment intact.
